# Exploration of Inbuilt Novel Properties as Bioretardant Cum Stabilizer of Isolated Biopolymer from *Fragaria ananassa* in Delivery of Nanosized Phenytoin

**DOI:** 10.22037/ijpr.2021.113734.14457

**Published:** 2021

**Authors:** Sushant Kumar, N.V. Satheesh Madhav

**Affiliations:** a *Faculty of Pharmacy, Pharmacy College Saifai, Uttar Pradesh University of Medical Sciences, Saifai, Etawah, U.P., 26130, India. *; b *Faculty of Pharmacy, DIT Univesity, Dehradun, U.K., India.*

**Keywords:** Biopolymer, Bionanoparticles, Bionanosuspension, Epilepsy, Fragaria ananassa, nanosizing, Phenytoin

## Abstract

The research aims to develop bionanosuspension using a biopolymer isolated from *Fragaria ananassa* fruit that constitutes potential and natural polymeric properties. At first, the biomaterial was isolated from the natural fruit pulp of *Fragaria ananassa* by an economical method of isolation. The model drug was nanosized by a novel sonication method. The isolated biopolymer was characterized for its polymeric properties, and its potential capabilities were evaluated in the delivery of nanosized phenytoin. The isolated biopolymer was characterized for DSC, FTIR, NMR, mass, and scanning electron microscopy. The isolated biomaterial was used for the preparation of phenytoin-loaded bionanosuspension with other excipients. The bionanoparticles were also characterized by different analytical testing such as FTIR, DSC, and SEM to confirm any interaction between model drug and biopolymer.

The prepared bionanoparticles showed the release of phenytoin in a sustained manner over 36 hours. The release kinetic study was done using the BIT-SOFT 1.12 software and other parameters such as t50%, t80%, and *r*^2^ were calculated. The formulation PFr6 was considered best having t50% in 18.22 hours and t80% in 29.62 h with an r^2 ^value of 0.9793. This formulation showed up to 87.89% drug release within 36 hours. The prepared bio-nanosuspension was found to be stable and in a well-dispersed state. The dried bionanosuspension evaluation revealed no interaction between model drug and biopolymer without any loss of characteristic peaks. Therefore, the isolated biopolymer can be safely used to prepare stable bionanosuspension loaded with nanosized phenytoin.

## Introduction

Polymers are the first choice for designing novel drug-delivery systems for sustained release, controlled release, extended release, and targeted drug-delivery systems (1).

Nowadays, researchers have proved that biopolymers are one of the most novel and intelligent biomaterials isolated from various natural sources such as bark, seeds, flowers, fruits pulps, leaves, and other natural sources (2). These biopolymers have good film-forming, mucoadhesivity, sustainability, retardability, release rate, and controlling ability. These can be used to design novel drug-delivery and targeted drug-delivery systems (3–5).

 In this research work, bionanosuspension with bionanoparticles loaded with nanosized phenytoin has been prepared using the novel biomaterial (7) from fruit pulp of *Fragaria ananassa*. For developing bionanoparticles and their suitability for the release of phenytoin, the potentiality of biopolymers was evaluated.

Epilepsy is a very common neurological disorder of the brain. In epilepsy, the brain does not normally act that results in seizures or episodes of abnormal behavior, sensations and may sometimes lead to loss of awareness (6). Phenytoin is an antiepileptic drug available in the form of tablets, capsules, and an oral suspension. By designing the antiepileptic drug-loaded bionanosuspension for targeting to brain via ear, the challenges in treatment of epilepsy on oral or other rote administration can be conquered easily, that too in an efficient way (7).

Zhou et al. researched liposomes coated with phenytoin prepared by thin-film dispersion method (8). These prepared liposomes were partially inhibitors of I/R injury and reduced the infarct size and left ventricular fibrosis after I.V. administration. Fuller *et al.* described the comparison of tolerability, safety measures, and different side effects of levetiracetam and phenytoin after intravenous administration (9). So in a regimen of epilepsy, no significant difference was observed between levetiracetam and phenytoin after intravenous plus oral regimen. Wang *et al.* developed electroresponsive hydrogen nanoparticles for the delivery of phenytoin in the brain (10). Phenytoin was distributed significantly to brain tissues and during tonic-clonic seizures. It was released in significant concentration for the treatment of seizures. Baumgartner *et al.* designed phenytoin-loaded nanosuspension (11), while Motawea et al. developed phenytoin-loaded nanostructured lipid carriers for topical delivery (12). In another study, Senthilvel *et al.* developed capsules filled with phenytoin- and berberine-loaded nanoparticles to improve anticonvulsant activity (13). In this research, biopolymer was isolated from the natural fruit pulp of *Fragaria ananassa* because of its excellent nutritional properties such as consistency in the rich amount of proteins, vitamins, and minerals (14), several industrial applications for the preparation of jam, jelly, and squash. It is also used in the cosmetic industry for preparing different products (15). Biopolymer has excellent film-forming ability and biostability properties (16, 17).

Using the biopolymer, a drug may be delivered in a retardant manner by utilizing its inbuilt bioretardant cum stabilizer properties (17). In this research work, the naturally isolated and characterized biopolymer can be used as an alternative to standard available polymers for targeting CNS for treat disorders associated with brain (18, 19). The drug solubility problems of the drugs can be minimized by converting the drug into its nanosized form (20). Nanosuspension provides an enhanced bioavailability, enhanced uptake by the cells, prolonged duration of action, and avoiding unwanted toxicity systemically (21). By designing the drug-loaded bio-nanosuspension with biopolymer, the solubility problems of the drugs can be solved (22). Mohammadi *et al.* developed whey protein isolated–based biodegradable films incorporated with chitosan nanofiber and cinnamon essential oil. It was concluded that a bio-nanocomposite in packaging technologies at the industrial level is necessary for improving the shelf life of food materials (23).

In this way, this research concludes the isolation of biopolymer from natural source *Fragaria ananassa* and use of isolated biopolymer in developing bionanosuspension for delivery of phenytoin significant as it is the prime need in the treatment of epilepsy for long term. Thus the novel inbuilt properties of isolated biopolymers are not explored yet as a novel drug delivery in pharmaceutical sciences. The biopolymer with novel properties such as bioretardant cum biostabilizer and its isolation at a very economical level can prove a magical key role in designing targeted drug-delivery systems. It can be used as an alternative to existing standard polymers (24).

## Experimental

*Materials*

Phenytoin was obtained as a gift sample from Affy Pharma Private Limited, Baddi. The *Fragaria ananassa* (strawberry) was purchased from the local market of Lucknow. All other chemicals used were of analytical grades, such as sodium benzoate (Thermo Fischer India Pvt. Ltd), dextrose (Thermo Fischer India Pvt. Ltd), and polyvinyl alcohol (Thermo Fischer India Pvt. Ltd.).

*Methods*

*Isolation of Biopolymer*

The *Fragaria ananassa* fruit was purchased from the local market of Lucknow. A volume of 500 g fruit pulp was washed with distilled water. Then it was ground in a grinder till it converted into a paste. A small quantity of distilled water can be added during the grinding, if necessary. This paste was filtered through the muslin cloth. The collected filtrate was centrifuged at 5000 rpm for 10 min (17). After centrifugation, the supernatant was taken. Centrifugation was performed to remove any residue. Then half of the supernatant was treated with methanol in a 1:1 ratio. These were placed in a refrigerator overnight. Then after treatment, these were centrifuged again at 5000 rpm for 30 min. The supernatant was discarded and the biomaterial as sediment was collected and air-dried. If there is any moisture content, it can be dried in desiccators for 48 hours. If the biomaterial (3) consists of any oil, it can be removed by washing with acetone or chloroform. This procedure was repeated and optimized six times, and the yield was calculated. The obtained biomaterial was passed through sieve number 200 and stored for further use (20).

*Characterization of Isolated Biopolymer*

The physicochemical properties of isolated biopolymer were as follows: color, odor, taste, and solubility. Chemical tests were also performed for the presence of carbohydrates, starch, and proteins. The isolated biopolymer was also characterized based on SEM analysis, DSC testing, IR spectroscopy, mass spectroscopy, and NMR spectroscopy.

*Chemical test of isolated biomaterial*

*Chemical test for carbohydrate*

An amount of 1 mL biopolymer solution (5% biopolymer solution in distilled water) was taken in a test tube. Then two drops of Molisch reagent were added, followed by 1–2 mL of addition. Sulfuric acid was poured into the test tube and observed until the purple color appeared at the interface of the two layers formed. The test was performed and reported (25)

*Test for the presence of protein*

For testing the presence of protein, the isolated biomaterial was treated with 0.1% solution of Ninhydrin reagent and 10% tannic acid solution. The presence of blue and yellow color precipitate indicates the presence of protein. The test was performed and reported.

A Biuret test was performed for the confirmation of proteins. A volume of 2 mL biopolymer was taken in a test tube (5% biopolymer solution in distilled water), and 1 ml of sodium hydroxide solution was added along with copper sulfate solution drops. The mixture was kept aside for 5 min, and the color changes were observed. The emergence of violet color confirms the presence of proteins. The test was performed and reported (25).

*Spectral analysis of the biomaterials*

Spectral analysis such as IR, NMR, mass spectroscopy, and SEM studies of the isolated biopolymer was conducted. The biomaterials were subjected to IR, NMR, mass spectroscopy studies, and the obtained spectra were interpreted and reported. Apart from that, SEM studies of different biomaterials were also performed, and the obtained results were interpreted and reported. IR, NMR, and mass spectroscopy were studied at Central Drug Research Institute Lucknow, and SEM studies were studied at Birbal Sahani Institute of Paleobotany Lucknow (26).

*SEM (scanning electron microscopy) analysis*

The isolated biopolymer’s surface morphologies were characterized by a scanning electron microscope. In SEM analysis, the external surface and internal structure were characterized. The small quantity of biopolymer was taken and fixed on aluminum studs and then coated with gold using the sputter under vacuum atmosphere by a gold sputter module in a high vacuum evaporator. The scanning electron micrograph was taken for the biopolymer under observation. SEM testing was performed at BSIP, Lucknow using Make, JEOL India Pvt Ltd. Model, JEOL JSM 7610F. The SEM images were interpreted and reported (26).

*FTIR spectroscopy of isolated biopolymer*

The FTIR spectroscopy was done by preparing the KBr discs. An amount of 1 mg isolated biopolymer was taken and mixed with 100 mg of dried and desiccated solid KBr. The mixture was mixed in mortar and pestle and placed in an IR lamp to remove any moisture. The mixture was converted into a disk under the pressure of 10 tons. The prepared disk was placed in a disk holder in the path of IR radiation. The spectrum was recorded within a range of 4000–200 cm^-1^. FTIR of hydroxypropyl methylcellulose was also carried out for characterization. The FTIR was done at CDRI, SAIF, Lucknow using Model-Agilent Cary 630 FTIR Spectrometer (range: 4000–450 cm^-1^). The FTIR spectra were interpreted and reported (26).

*Mass spectroscopy*

It is a useful and powerful technique for quantifying known materials and identifying unknown materials and elucidating the structure and chemical properties of the molecule. It is an accurate method for determining the molecular mass of the compound. This is performed in a laboratory in which the sample of biopolymers was introduced through the inlet system. The gas-phase ions of the compound were produced. Then with the help of molecular ion fragmentation, the ions were separated in mass spectrometer according to their mass-to-charge ratio. A mass spectrum of ion abundance versus mass-to-charge ratio was recorded, which was done at CDRI-SAIF Department, Lucknow by using Waters PLC-TQD mass spectrometer (ESI-MS, APCI-MS, and LC-MS/MS) and Agilent 6520 Q-TOF (ESI-HRMS and APCI-HRMS). Mass spectra were interpreted and reported (26).

*NMR spectral analysis*

The NMR spectroscopy was done for spectral analysis of isolated biopolymer. The sample was dissolved in a specific solvent such as CDCl_3_. The mixture was pumped into the instrument at a high rate of flow. The switch for the valve was used to stop the flow. Then the measurement was performed. After the finishing of measurement, the spectrum was processed and analyzed in an automation computer. This was done at CDRI, SAIF, Lucknow using Advance-400 MHz Bruker, Switzerland. NMR spectra were interpreted and reported (26).

*Differential scanning calorimetry testing*

DSC testing is the thermal analysis technique in which the heat flow inside or outside of the sample for testing is determined as the function of temperature. Here, the biopolymer sample was taken and exposed to a controlled temperature program. The glass transition temperature was determined. The heat flow range was 30–300 °C. The DSC thermogram was recorded, interpreted, and reported (26).

*Nanosizing of phenytoin by sonication method*

A volume of 500 mg phenytoin was taken and dissolved in 25 mL methanol. The clear solution was sonicated for 15 cycles continuously. During sonication, 25 mL of purified water was added drop by drop till precipitation was observed. The obtained precipitate was allowed for centrifugation. After each sonication cycle, the sample was allowed for absorbance and %transmittance (%T) and %blockage (100‒%transmittance) measurement. The residue was recovered and then dried to collect the nanosized phenytoin in the nanoparticles range. This nanosized phenytoin obtained by this standard solvent evaporation method was evaluated for different parameters. The dried nanosized phenytoin was packed and stored for further use. The phenytoin was also nanosized by a novel sonication method. Here 500 mg of phenytoin was taken and mixed with dextrose and 25 mL double distilled water. This dispersed solution was sonicated for 15 cycles continuously. During sonication, 25 mL of purified water was added drop by drop till precipitation was observed (17). The obtained precipitate was allowed for centrifugation. After each sonication cycle, the sample was allowed for absorbance and transmittance% (T%) and blockage% (100‒transmittance%) measurement. The residue was recovered and then dried to collect the nanosized free-flowing phenytoin powder. The procedure was repeated in triplicate.

*Drug–Excipient Interaction Study*

The drug–biopolymer interaction study was performed by the UV spectroscopy method. The phenytoin–biopolymer mixture was prepared in a ratio of 1:1, 1:3, and 3:1 by wet and dry mixing. After mixing, the drug and polymer mixture was preserved at a temperature of 50 °C for 3 days in the wet method and then diluted with solvent and scanned for the absorption maxima (***λ***_max_). In the dry method, three different ratios of drug–biopolymer were prepared in their physical form. Then after storing at room temperature, this was diluted with 2 mL of methanol and then scanned by UV spectrophotometer for any change in ***λ***_max_.

*Formulation of Phenytoin-Loaded Bionanosuspension*

The formulations of bionanosuspension were prepared using the different drug–biopolymer ratios, as shown in Table 1. The bionanosuspension was prepared by sonication of the mixture of drug and biopolymer and other excipients such as polyvinyl alcohol as a suspending agent, sodium benzoate as the preservative, purified water, and dextrose as nanosizent. The phenytoin, Fragaria × ananassa biopolymer, and other excipients were accurately weighed and triturated with double-distilled water. This mixture was sonicated for 3 cycles. Then 0.5 mL of polyvinyl alcohol (0.5%) was added during sonication. The volume was made up to 10 mL with double-distilled water having sodium benzoate content of 0.1–0.5%. Dextrose can be added if necessary as a nanosizing agent and allowed for sonication for 15 cycles. After sonication, the bionanosuspension was refrigerated for 2 days. If there is no settlement, then it can be considered that the formulation is optimized, but if having a settlement, then the abovementioned amount of polyvinyl alcohol was again added and allowed for sonication for 10 cycles and refrigerated for 48 h. Different formulations were prepared, and after optimization according to stability, the formulations PFr1–PFr6 were prepared. After formulation, their stability was tested and then evaluated for different parameters, including release study (20).

*Characterization of phenytoin-loaded bionanosuspension*

*Dispersibility study of bionanosuspension*

A volume of 10 mL formulated bionanosuspension was taken and dispersed in 20 mL of the distilled water in a test tube. The time for settling of the dispersed nanoparticles in the bottom was noted, and then again, the nanoparticles were redispersed and noticed for the redispersion. After shaking, any lump or aggregates or any precipitation formation was observed (20). The procedure was repeated in triplicate.

*pH study of bionanosuspension*

The pH of formulated bionanosuspension was evaluated with a digital pH meter. The study was done in triplicate, and the mean was taken and checked which helps determine if the pH of the nanosuspension is within the required range. The procedure was repeated in triplicate (20).

*Entrapment% efficacy of loaded bionanosuspension*

The freshly formulated bionanosuspension was taken and centrifuged at 5000 rpm in an ultracentrifuge. After centrifugation, the supernatant was taken and diluted up to 10 µg/mL, and the amount of drug unincorporated was measured by determining the absorbance under UV spectroscopy at 216 nm. The amount of the drug loaded in the nanoparticles was calculated by subtracting the amount of free drug in supernatant from the initial amount of drug taken in the formulation. The procedure was repeated in triplicate (22). This determination was done in triplicate, and the average was calculated by using the following formula:

$\mathrm{Entrapment efficacy\%}=\frac{\mathrm{Amount of drug loaded in nanoparticles}}{\mathrm{Initial amount of drug taken in formulation}}\times 100$

*Particle size screening of the nanoparticles in bionanosuspension by UV method*

The bionanosuspension was evaluated by measuring the percent transmittance of the bionanosuspension. The percent transmittance was measured as a function of the particle size in nanorange done by the sonication method. The % transmittance depends on the particle size range within a particular range that defines the size of the particles below or beyond the size range required. The transmittance% was determined before and after the sonication cycle. The transmittance% at different wavelengths indicates that when the light passes through the particles, it indicates that the particle size is below that wavelength which shows that the percentage of the particles is less than 400 nm in the mixture and the blockade% shows that the percentage of particles is more than that. The transmittance% was measured by using the UV spectrophotometer. After each sonication cycle, it was increased due to the reduction of the particles to nanorange. The effect of sonication on %transmittance was observed after sonication and measuring after each sonication cycle (22). The procedure was repeated in triplicate.

*Particle Size Analysis*

The particle size of the bionanosuspension was studied by characterizing with the Malvern Zetasizer. The particle size distribution by intensity was confirmed by using the zetasizer (22).

*In-vitro release study of bionanosuspension*

The in-vitro release study was performed for all formulations. *In-vitro* release study was performed by a novel static method by using modified M.S. Diffusion apparatus. It consists of two compartments: one for the donor and the other for the receiver. The formulation for the release study was taken in the donor compartment (1 mL) and the end of the donor is tied with the egg biomembrane. This donor compartment was immersed in the receiver compartment having 13 mL of pH 7.4 phosphate buffer solution. Sampling was done at different regular time intervals for 36 h. The samples were withdrawn completely and replaced with the fresh phosphate buffer solutions after every sampling. The samples were analyzed by UV spectrophotometer for determining the released amount of the drug. The graph was plotted between the CDR% and time. Other parameters such as r^2^, t50, and t80% were calculated to evaluate release study from different formulations and the selection of the best formulation (22).

*Stability Study*

The stability study was performed as per ICH guidelines. The formulated bionanosuspension was stored for 6 months at different ambient temperatures. The bionanosuspension was kept at two different conditions at 25 °C ± 2 °C, 60% RH and 40 °C ± 2 °C, 75% RH stability chamber. The samples were observed every 2 weeks for their different parameters during the testing period. The samples under evaluation were observed for the drug content, pH changes, any changes in color, appearance, entrapment efficacy (22).

## Results and Discussion

*Isolation of Biopolymer*

The *Fragaria ananassa* biopolymer was found to be white with a yield% of 13 ± 2. The color-changing point was found to be 229 °C ± 5 °C.

*Characterization of Isolated Biopolymer of Fragaria ananassa*

The isolated biopolymer was white color in appearance. The biopolymer was found to be odorless with a characteristic taste. It was found to be sparingly soluble in water. It showed a positive test for carbohydrate and protein (20). The characterization of isolated biopolymer of *Fragaria ananassa* is shown in Table 2.

*SEM analysis of biopolymer*

**Figure 1:**
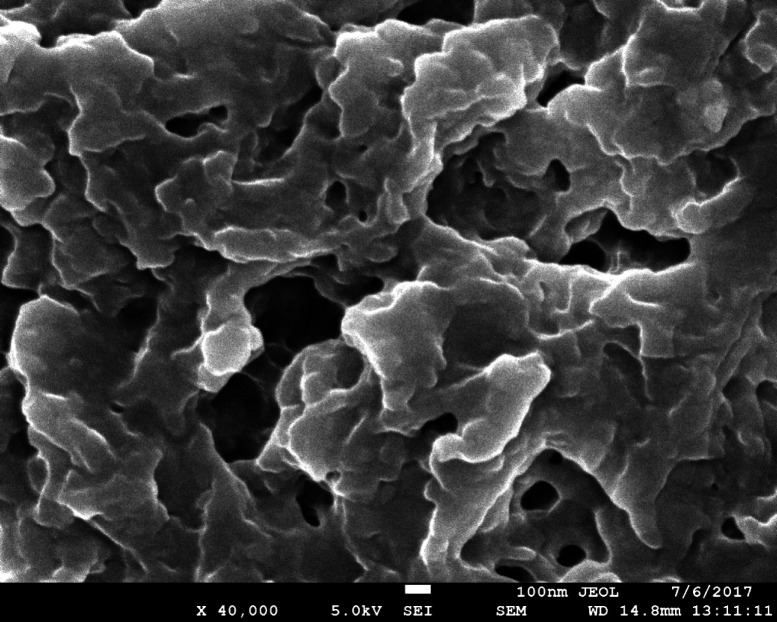
SEM of isolated biopolymer from *Fragaria ananassa* at 40,000×.

The isolated biopolymer was analyzed by scanning electron microscopy for surface characterization. The SEM analysis shows the rough and flaky structure of the biopolymer. The granular structure was also observed in the SEM image. This confirms the polymeric nature of the biopolymer having a flaky and granular structure (22). The SEM image of isolated biopolymer is shown in Figure 1.

*Different Spectral Analysis and Their Findings*

*IR spectral analysis of isolated biopolymer*

**Figure 2:**
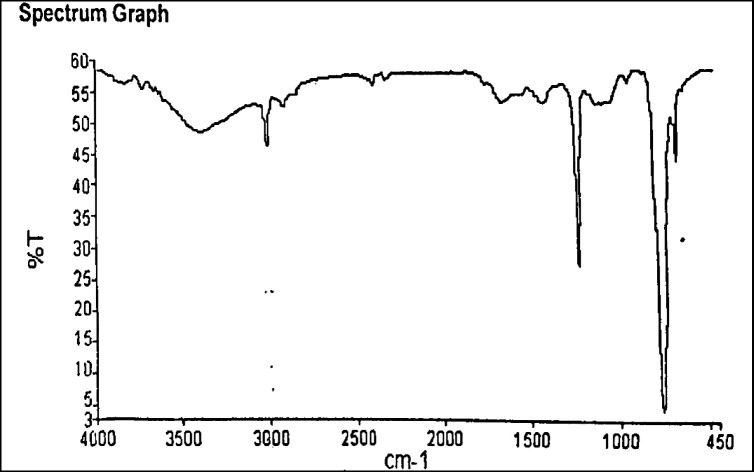
FTIR spectra of biopolymer from *Fragaria ananassa*

FTIR spectra of the isolated biopolymer show the presence of different functional groups responsible for the polymeric nature of the isolated biopolymer. The IR spectra show the presence of different functional groups such as hydroxyl (3396.15 cm^-1^), alkynes (669.43 cm^-1^), and carboxylic acid (1410.35 cm^-1^), which confirms its polymeric characteristics. Other groups such as amide at 1639.02 cm^-1^, alkane at 2925.45 cm^-1^, tertiary alcohol at 1215.89 cm^-1^ were present in the IR spectra. The presence of these functional groups is responsible for the retardibility in drug release like other standard polymers (22). FTIR spectra are shown in Figure 2.

*Differential scanning calorimetry (DSC) study of isolated biopolymer*

**Figure 3:**
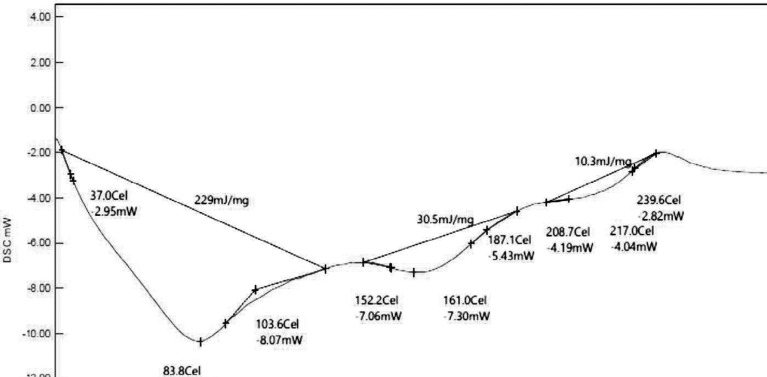
DSC of biopolymer from *Fragaria ananassa*

The DSC thermogram of *Fragaria ananassa* shows peaks at 83.794 Celsius, 161.035 Celsius, and 208.709 Celcius. The area was found to be 229 mJ/mg, 30.5 mJ/mg, and 10.3 mJ/mg, respectively. The thermogram showed the sharp endothermic broad peak that deals with the amorphous nature of biopolymer (Figure 3).

*Mass spectroscopy of isolated biopolymer*

**Figure 4:**
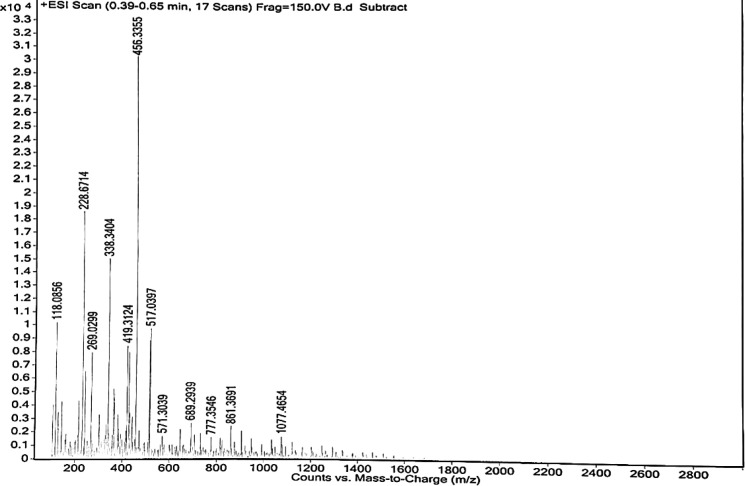
High-resolution mass spectrum of isolated biopolymer from *Fragaria ananassa*

Mass spectra reveal that the isolated biopolymer is polymeric due to the presence of large molecular weight structures. It indicates the presence of protein. HRMS spectra of isolated biopolymer showed the parent peak at m/z 456.33, confirming its large molecular weight structure such as a polymer (27) (Figure 4).

*NMR spectroscopy of isolated biopolymer*

**Figure 5:**
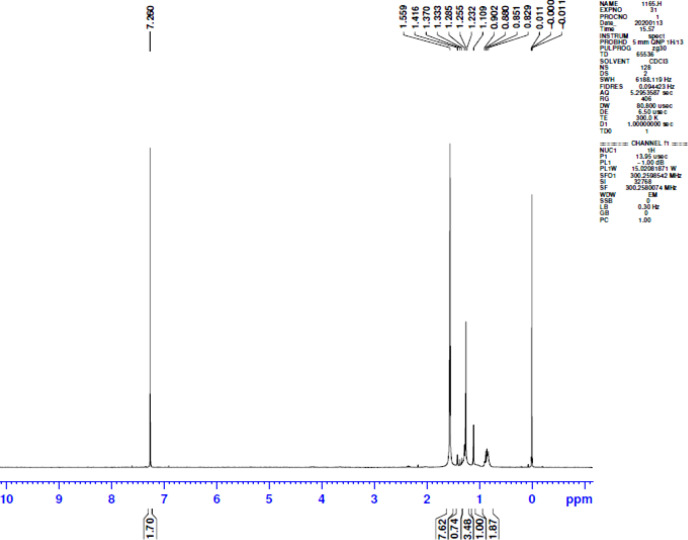
NMR spectra of biopolymer from Fragaria × ananassa

The NMR spectra show the presence of different peaks such as multiplet at 0.829–0.902 ppm which reveals the presence of primary alkyl group; peaks at 1.232 ppm confirm the presence of methylene group and at 1.255 ppm the presence of hydroxyl group. This presence confirms its polymeric nature (27) (Figure 5).

*Nanosizing of phenytoin*

During the nanosizing of phenytoin after each sonication cycle, the sample was observed for measurement of percent transmittance which confirmed that when the number of sonication cycle was increased, the percent transmittance was also found to be increased. This was due to a decrease in particle size and particles are now are in nanorange. Thus, transmittance percent shows that the percentage of particles less than 400 nm in bionanosuspension and blockadepercent gives an idea about the percentage of particles which is above 400 nm. Thus the UV method has given an idea about particles in nanorange (22).

*Drug-Excipient Interaction Study*

No change was noticed in ***λ***_max_ before (216 nm) and after the test (216 nm) in the drug-excipient study. The absorption maxima of phenytoin *Fragaria ananassa* mixture was closed to the ***λ***_max_ of pure drug. There was no significant change in ***λ***_max_ of drug–polymer mixture as compared to pure drug. It means that it confirms no interaction between drug and biopolymer and other excipients too. It was observed that excipients are not interacting and not producing any changes in drug properties so that the isolated biopolymer can be used to prepare bionanosuspension (27).

*Formulation of Phenytoin-Loaded Bionanosuspension*

Different formulations of bionanoparticles by using different ratios of biopolymer from *Fragaria ananassa* and phenytoin were prepared. Then post formulation of bionanosuspension was evaluated for different parameters and their finding are described below.

*Dispersibility Study of Bionanosuspension*

The dispersibility of the formulated bionanoparticles was found to be excellent, and the redispersion was also found to be good. All nanoparticles were in a dispersed state during dispersion (20). No aggregation or lump formation was observed (Table 3).

*pH study of Bionanosuspension*

The pH of the bionanosuspension was found to be in the range of 7.3 ± 0.22 to 7.7 ± 0.19. It means that the formulations were in desired pH range that is suitable for the stability of the bionanosuspension (20). The pH of the different bionanosuspension formulations observed is given in Table 3.

*%Entrapment efficacy of loaded bionanoparticles*

The entrapment efficacy of the formulated bionanosuspension was found in the range of 84.56 ± 2 to 88.02 ± 1.8%. Thus the formulated bionanosuspension PFr6 showed the maximum entrapment efficacy up to 88.02 ± 1.8% (Table 3).

*Transmittance% of Bionanosuspension*

*Transmittance% *was found to be in the range of 91 ± 1.2% to 98 ± 0.75% after 15 cycles of sonication. Here, the UV method was used for screening the size of bionanoparticles in bionanosuspension. It was observed that as the sonication cycle was increased, the %transmittance was found to be increased because the particle size after sonication has come in nanorange. The transmittance% indicated the percentage of particles below 400 nm, and the blockade% showed the percentage of particles above 400 nm when screened by the UV spectrophotometry method. Thus, the UV method can be used as a screening method to determine nanoparticles’ size in bionanosuspension (20).

*Particle size analysis*

**Figure 6:**
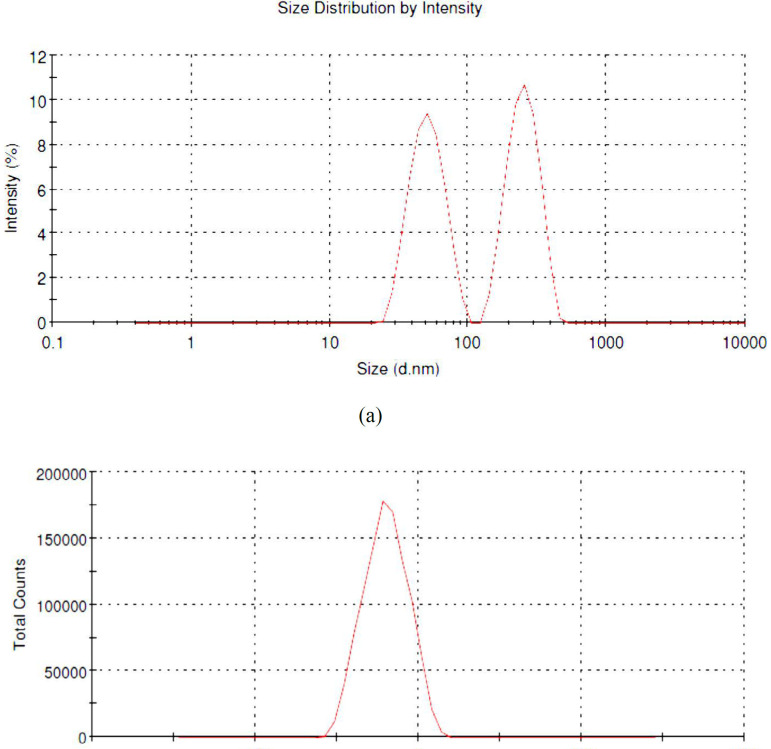
(a) Particle size distribution in bionanosuspension and (b) zeta potential

The nanoparticles size in bionanosuspension (PFr6) was 238.5 nm after evaluating with Malvern Zetasizer. Thus, the obtained size with the zeta potential of ‒20.1 mV confirms that the nanoparticles are in nanorange, which is responsible for the stability of nanosuspension. It also confirms that the stable bionanosuspension loaded with phenytoin was prepared using smart isolated *Fragaria ananassa* biopolymers. The result reveals that it can be safely used for delivering the phenytoin from the prepared bionanoparticles in the treatment of epilepsy (20). The particle size distribution in bionanosuspension is shown in Figures 6a and 6b.

*FTIR of bionanoparticles and phenytoin-loaded bionanoparticles*

**Figure 7:**
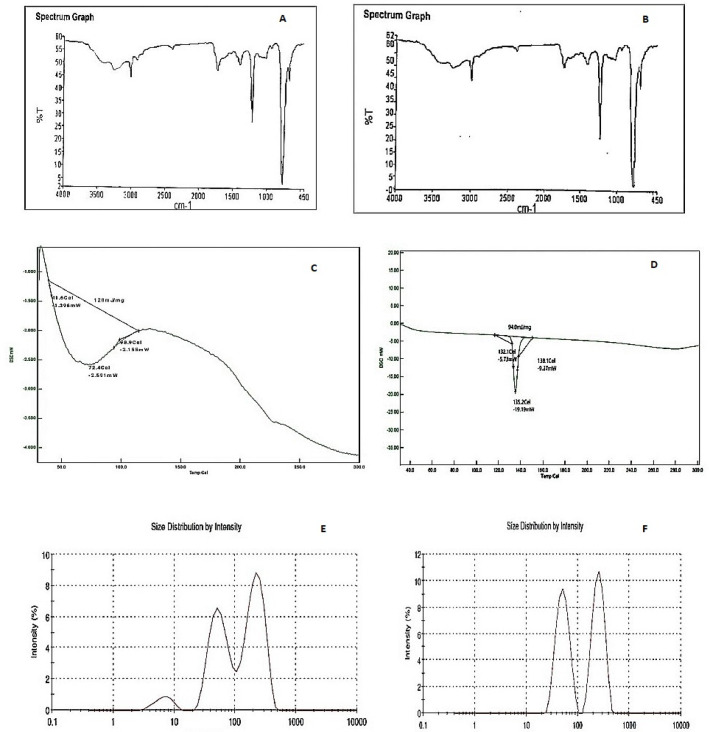
A. FTIR of bionanoparticles B. FTIR OF Phenytoin loaded bionanoparticles C. DSC of bionanoparticles D. DSC of Phenytoin loaded bionanoparticles E. 136.1 nm size of bionanoparticles F. 147.7 nm size of Phenytoin loaded bionanoparticles

The FTIR spectroscopy of the dried phenytoin-loaded bionanoparticles revealed no interaction between the model drug and the biopolymer used. The appearance of a new peak or disappearance of the existing peaks was not observed in this spectroscopy. The results indicated that there was no loss of the functional peaks of drug having C–H stretching, C–H bending, C–O stretching, and O–H stretching with isolated biopolymers with much-closed peak values as that of the pure drug, whereas FTIR of bionanoparticles suggested the biopolymeric nature of bionanoparticles with the presence of characteristic peaks very similar to the peaks as observed in FTIR of biopolymer at 669 (C–H bending), 1215.93 (C–O stretching), 3021.27 (C–H stretching) and 3264.99 cm^-1^ (O–H stretching). The obtained spectra of bionanoparticles and phenytoin-loaded bionanoparticles are shown in Figures 7A and 7B. 

*Differential scanning calorimetry of bionanoparticles and phenytoin-loaded bionanoparticles*

The DSC of bionanoparticles showed the biopolymeric nature with a broad endothermic peak at 72.4 °C. The DSC thermogram of phenytoin-loaded bionanoparticles revealed its crystallinity. These also showed a sharp endothermic peak at 135.2 °C which indicated the presence of a small and wide endothermic peak, further showing that the crystalline drug was converted in partially amorphous form during the nanosuspension formulation and nanosizing. There was also a shift of melting peak to the lower temperature in formulated bionanoparticles which was due to the conversion of phenytoin crystal form in nanorange during sonication. The obtained DSC thermogram of bionanoparticles and phenytoin-loaded bionanoparticles is shown in Figures 7C and 7D.

*Zeta particles size of bionanoparticles and phenytoin-loaded bionanoparticles*

The zeta particle size of bionanoparticles and phenytoin-loaded bionanoparticles analysis revealed that the bionanoparticles were found to be 136.1 nm and its size was found to be 147.7 nm (refer to Figures 7E and 7F). The bionanoparticles and phenytoin-loaded bionanoparticles zeta particle sizes were suitable for an easy target to the desired site.

*SEM of phenytoin-loaded bionanoparticles*

**Figure 8:**
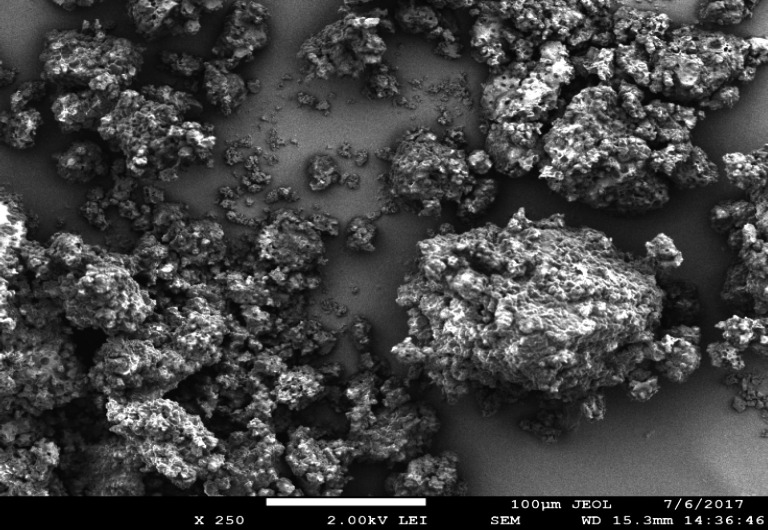
SEM of phenytoin-loaded bionanoparticles

The scanning electron microscopy of phenytoin-loaded bionanoparticles showed a more regular uniform shape with more or less rough surfaces. The aggregates were observed, which may result from the aggregation of some individual bionanoparticles because of the reveal of water or any moisture during the drying of bionanosuspension in the form of dried bionanoparticles. This more or less rough surface may be attributed to the adsorption and biopolymer coating of the model drug. SEM of phenytoin and bionanoparticles is shown in Figure 8.

*In-vitro release study of bionanosuspension*

**Figure 9:**
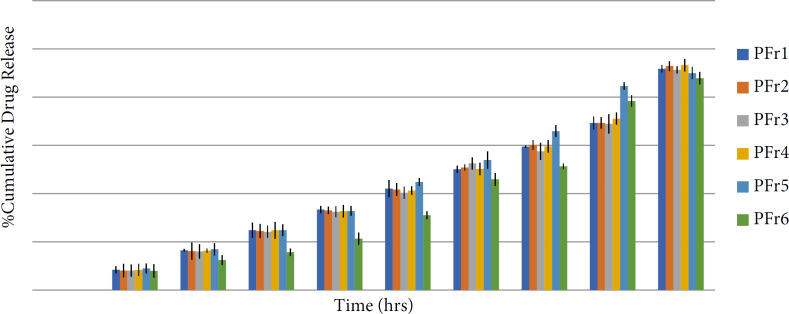
*In-vitro* release drug profile of different bionanosuspension formulations (PFr1–PFr6). The results are expressed as mean ± SD (n = 3)

The in-vitro release study was done using the M.S. Diffusion apparatus. The release kinetic study was done using the BIT-SOFT 1.12 software and other parameters such as t50%, t80%, and r^2^ were calculated. All the formulations showed more than 87.89% drug release (Figure 9). The *in-vitro* release study of different formulations showed the drug% release from 87.89 to 93.26. The different formulations were evaluated for the *in-vitro* release study and release kinetic was studied. The formulation PFr6 was the best formulation with t50% of 18.22 hours and t80% of 29.62 h with a r^2^ value of 0.9793. The best formulation -PFr6- showed up to 87.89% drug release in 36 hours. According to the release kinetic study, the best fit model was found to be Korsmeyer–Peppas and the mechanism of drug release was found to be anomalous transport. The result obtained from the *in-vitro* release study and analysis of the release kinetic of all formulations indicates the sustained release of the phenytoin from the bionanosuspension (22).

*Stability Study*

The optimized formulations showed no change in ***λ***_max_, entrapment efficacy and drug release. So there was no drug loss during the study period. The other evaluation parameters also showed satisfactory result. The best-optimized formulation was found to be stable over 6 months. There was no change in color, odor, pH, and physical appearance. During the stability study period, the results obtained were satisfactory from different parameters and the formulation PFr6 was found to be the best optimized stable formulation. The obtained results from the study confirmed that the formulation was physically and chemically stable (22).

The biopolymer is a novel biomaterial with many in-built properties that may be used for delivering drugs to the target. The isolated biopolymer may be used to prepare a novel and intelligent carrier system for loading phenytoin for the treatment of epilepsy. The bionanoparticles are the nanorange particulate systems that may efficiently deliver antiepileptic drugs such as phenytoin.

In this research work, phenytoin was nanosized by a novel and standard method. The biopolymer was isolated from the fruit of *Fragaria ananassa* with good polymeric properties. It can be suitably used for the preparation of bionanoparticles in the form of bionanosuspension. The isolated biopolymers showed good entrapment efficacy. This biopolymer has novel in-built properties such as filmability, retardability, and release rate controlling capability. It may be used for preparing suitable bionanosuspension for delivering nanosized phenytoin. In-vitro release and release kinetic study reveal that the isolated *Fragaria ananassa* biopolymer consists of the desired bioretardant and biostabilizer novel properties. The nanosized phenytoin particle size was screened by the UV method that gave an idea about the particle size range. This method may be used to screen of the nanosize range of phenytoin as well as prepared bionanosuspension (22).

The obtained results reveal that the isolated biopolymer consists of promising polymeric properties that can be used as a bioretardant cum stabilizer to prepare stabilized bionanosuspension. The spectral characterization reveals its polymeric nature (27).

The pH, dispersibility, and entrapment efficiency were found to be significant. The nanosizing of the drug showed satisfactory results for entrapment as well as in overcoming the solubility problem of phenytoin (20).

The particle size of the formulated bionanosuspension was evaluated by measuring the %transmittance as well as with the help of measuring zeta particle size and zeta potential (22).

The transmittance% measurement revealed that the prepared bionanosuspension has a satisfactory particle size in nanorange that is responsible for its stability. The particle size for the zetasizer was 238.5 nm, confirming its nanoparticle size range for the best formulation PFr6. The stability of bionanosuspension was also showed significant with ‒20.1 mV zeta potential. This means that the particles are in a well-dispersed state without any agglomeration with good repulsive force (22). 

Thus, bionanosuspension (PFr6) prepared using the biopolymer from *Fragaria ananassa* showed a significant entrapment efficacy and sustained release of phenytoin for more than 36 hours. So biopolymer from *Fragaria ananassa* can be safely used for the formulation of stable bionanosuspension (29, 30). The isolated *Fragaria ananassa* biopolymer was novel, nontoxic, nonreactive, biocompatible, inert, and biodegradable. So the biopolymer can be safely used as the novel biomaterial in delivering nanosized phenytoin (31). It can be safely used as an alternative to synthetic and semisynthetic available polymers for delivering phenytoin in the treatment of epilepsy.

The phenytoin-loaded bionanosuspension was converted into dried bionanosuspension. The dried bionanoparticles evaluated by different instrumental techniques revealed no loss of functional groups during the preparation of bionanosuspension. The biopolymer was found to be compatible with the drug. The evaluation findings revealed that *Fragaria ananassa *could be safely used to isolate of biopolymer and its utilization in preparation phenytoin-loaded stable bionanosuspension. The stability of bionanosuspension as well as bionanoparticles, is attributed to its inbuilt biostabilizing property. The release of drug from bionanoparticles revealed that biopolymer could also be safely used to develop bionanoparticles for the release of model drug in a sustained manner for a prolonged time, which is the prime need for long-term treatment of epilepsy. Thus, biopolymer isolated from *Fragaria ananassa *can be safely used in developing novel bionanosuspension.

**Table 1:** Formulation table of phenytoin-loaded bionanosuspension using *Fragaria ananassa* biopolymer

**Formulations**	**PFr1**	**PFr2**	**PFr3**	**PFr4**	**PFr5**	**PFr6**
Drug:biopolymer ratio	1:4	1:5	1:8	1:10	1:12	1:15
Phenytoin (mg)	10	10	10	10	10	10
*Fragaria ananassa*	40	50	80	100	120	150
Biopolymer (mg)						
Polyvinyl alcohol (mL)	0.5	0.5	0.5	0.5	0.5	0.5
Sodium benzoate (%)	0.5	0.5	0.5	0.5	0.5	0.5
Double-distilled water (mL)	10	10	10	10	10	10

**Table 2:** Characterization of isolated biopolymer of *Fragaria ananassa*

**Parameters evaluated**	**Observation**
Color	White
Odor	Characteristic
Taste	Characteristic
Melting Point	229 C ± 5 C
Solubility	Soluble in water, soluble in methanol
Carbohydrate	Present
Protein	Present

**Table 3:** Different formulations with observed pH, dispersibility, entrapment% efficacy values

**Formulations**	**Observed pH**	**Dispersibility**	**Entrapment efficacy (%)**
**PFr1**	7.4 ± 0.32	+	84.56 ± 2.0
**PFr2**	7.3 ± 0.22	+	86.16 ± 2.8
**PFr3**	7.5 ± 0.11	+	85.16 ± 1.6
**PFr4**	7.7 ± 0.19	+	86.20 ± .88
**PFr5**	7.4 ± 0.12	+	87.02 ± 1.8
**PFr6**	7.5 ± 0.05	+	88.02 ± 1.8

## Conclusion

This research work concludes that the isolated biopolymer from *Fragaria ananassa* fruit serves as a potential, natural polymeric nature biomaterial that can be used for preparing nanosized phenytoin-loaded bionanoparticles in the form of bionanosuspension. The best formulation PFr6 prepared using this natural and biodegradable isolated biopolymer from the edible natural fruit pulp was stable. It releases a significant amount of nanosized phenytoin for the treatment of epilepsy (32). So it was concluded that the isolated biopolymer from *Fragaria ananassa* fruit might be safely used as the novel biomaterial in delivering nanosized phenytoin from the formulated bionanoparticles significantly for an extended time (33).
